# Evaluation of surgical evacuation of the uterus for abortion and management of miscarriage in patients with sickle cell disease: A multicentre UK study

**DOI:** 10.1111/bjh.70449

**Published:** 2026-03-26

**Authors:** Sonia Wolf, Natasha Lewis, Funmi Oyesanya, Karlene Birthwright, Tracey Masters, Andriana Andreeva

**Affiliations:** ^1^ Barts Health NHS Trust London UK; ^2^ NHS Blood and Transplant London UK; ^3^ Blizard Institute, Queen Mary and Westfield London UK; ^4^ Homerton Hospital London UK; ^5^ Barking, Havering and Redbridge Trust London UK

**Keywords:** obstetric, pregnancy, sickle

To the Editors,

As standards of care improve for patients with sickle cell disease (SCD), more patients with SCD can plan and make choices for their reproductive years. Many authors have reported outcomes of completed pregnancy in SCD, but there is less evidence around abortion and miscarriage, as many studies exclude pregnancies ending before 24 weeks. Studies from the United States reported miscarriage rates of 15%–30%,^1^, ^2^ with similar rates in the United Kingdom.^3^ Abortion rates in SCD may be guided by local legalities, with reported rates of 6% in the United States,^1^ but UK‐specific data are lacking. In general, there are 20.6 abortions/1000 women aged 15–44 per year in England and Wales^4^ making it one of the most common surgical procedures in this demographic. Despite this, a lack of knowledge of the management of unplanned pregnancy in SCD was highlighted in a recent review.^5^

While many miscarriages and abortions are managed medically, surgical management may be preferential in SCD, as severe anaemia may require extra consideration and planning.^6^ Surgical abortion and surgical management of miscarriage (SMM) usually take place as day case procedures, under general anaesthetic of a short duration, typically less than 30 min or regional anaesthesia with a para‐ or intra‐cervical block. The procedure, typically vacuum aspiration for up to 14 weeks of gestation, is the same for abortion and SMM. Guidelines for transfusion for low‐ and medium‐risk surgical procedures largely recommend transfusion pre‐operatively, to avoid post‐operative complications, such as acute chest syndrome, which is higher without pre‐operative transfusion.^7^ However, there were no cases of surgical abortion or SMM in the original Transfusion Alternatives Preoperatively in Sickle Cell Disease (TAPS) study.

Two recent studies have looked at abortion and SMM in the United States. In a study of 21 patients undergoing surgical abortion or SMM, none were transfused pre‐procedurally.^8^ The second reviewed abortions and SMM across two settings, with around half occurring in outpatient clinics and half occurring in the operating theatre.^9^ While anaesthetic data were not reported in the latter, there is a suggestion that those done in the operating theatre were performed under general anaesthesia. This differs from the United Kingdom in two respects: first, SCD is generally considered a significant co‐morbidity and any patients disclosing this to independent sector providers are referred to National Health Service (NHS) settings.^10^ Second, surgical evacuation of the uterus is commonly carried out using regional and local anaesthesia in many cases. In addition, different models of healthcare may mean that the UK and other publicly funded healthcare systems do not place a financial burden on the patient and influence decision‐making around aspects of care, such as the need for transfusion or inpatient admission.

This service evaluation was carried out in two specialist hospital settings that perform surgical evacuations of the uterus and have specialist haemoglobinopathy services on‐site. We identified abortions and SMM in SCD patients, using medical coding records between 2013 and 2025. Records were searched using the terms ‘Vacuum aspiration of products of conception’ and ‘Dilation of cervix uteri and evacuation of products of conception from uterus’ and ‘sickle cell disease’ or ‘sickle cell anaemia’. Identified records were confirmed manually by the research team. Data were collected on patient demographics, sickle genotype, haematological and obstetric history, as well as procedure details, prior transfusion and any surgical or medical complications post‐procedure. Statistical data were compiled using R Studio version 4.3.1.

We identified a total of 53 procedures carried out in 44 women across the study period: 36 abortions, of which 33 were performed surgically and three were performed medically but required subsequent surgical intervention for retained products of conception, and 17 SMM. Demographic data are presented in Table 1.

**TABLE 1:** Demographic and past medical history data from all procedures.

	All (range) (procedures = 53, patients = 44^b^)	Surgical abortion^c^ (procedures = 36, patients = 30)	SMM (procedures = 17, patients = 15)
Age (median, range)	29 (16–42)	26 (16–35)	35 (25–42)
Ethnicity (*n*, %)^a^			
Black African	22 (50)	12 (40)	10 (67)
Black Caribbean	7 (16)	6 (20)	1 (7)
Black Other	10 (23)	7 (23)	3 (20)
Mixed	1 (2)	1 (3)	0
Asian	1 (2)	1 (3)	0
Other	3 (7)	3 (10)	0
Genotype (*n*, %)^a^			
HbSS	32 (73)	25 (83)	7 (47)
HbSC	10 (23)	3 (10)	7 (47)
HBSβ‐thal	2 (5)	2 (7)	0
History of acute^a^ chest syndrome (*n*, %)	2 (5)	1 (3)	1 (7)
Disease‐modifying therapy at time of procedure (*n*, %)			
Transfusions	14 (26)	8 (22)	6 (35)
Hydroxycarbamide	3 (6)	2 (6)	1 (6)
Voxelotor	1 (2)	1 (3)	0 (0)
None	35 (66)	25 (69)	10 (59)
Hospital attendances in past 12 months for sickle‐related issues (median, range)			
ED/medical day unit only	1 (0–50)	1 (0–50)	1 (0–20)
Hospital admission	0 (0–16)	0 (0–12)	0 (0–16)
Previous pregnancies (median, range)	1 (0–6)	1 (0–6)	1 (0–5)

Abbreviation: SNM, surgical management of miscarriage.

^a^ Percentages represent for constant variables (ethnicity, genotype, history of acute chest syndrome) are calculated using total number of patients as the denominator. All other results are calculated per procedure.

^b^ Please note total patient numbers from the SMM and surgical abortion columns add up to 45 as one patient had two surgical abortions and one SMM.

^c^ Total number includes three cases of medical abortion requiring surgical evacuation of the uterus afterwards.

Of 36 abortions, 31 (86% of total procedures) occurred in the first trimester, while five were in the second trimester (at 15/40 [*n* = 2, 6%], 16/40 [*n* = 2, 6%] and 17/40 [*n* = 1, 3%]). Sixteen of 17 SMM (94%) occurred in the first trimester, with one performed at 16/40 (6%). Procedural data are presented in Table 2. Of 53 procedures (both SMM and TOP), 33 (62%) were performed under general anaesthesia, while 19 (34%) were performed using local/regional anaesthesia and one was unknown. The median Hb prior to all procedures was 88 g/dL. Fourteen procedures (26%) were performed with prophylactic red cell transfusion (up to 72 h prior to the procedure). The median number of units transfused was 2 (range 1–3). There was one mild transfusion reaction (fever). The median Hb prior to transfusion was 82 g/L and post‐transfusion was 91 g/L.

**TABLE 2:** Procedural data from all surgical evacuation of the uterus procedures.

	All (range) (procedures = 53, patients = 44)	Surgical abortion (procedures = 36, patients = 30)	SMM (procedures = 17, patients = 15)
Procedure type (*n*, %)^a^			
Surgical abortion	33 (62)	33 (92)	
Surgical evacuation of the uterus following medical abortion	3 (6)	3 (9)	
Surgical management of miscarriage	17 (26)		17 (100)
Median gestation (completed weeks)	9 (17)	9	10
Pre‐operative Transfusion	14 (26)	9 (25)	5 (29)
Anaesthesia			
General	33 (62)	24 (67)	9 (53)
Local/regional	19 (36)	11 (31)	8 (47)
Unknown	1 (2)	1 (3)	0
Surgical complications			
Haemorrhage (EBL >500 mL)	2 (4)	1 (3)	1 (6)
Uterine perforation	0 (0)	0 (0)	0 (0)
Endometritis	0 (0)	0 (0)	0 (0)
Post‐operative transfusion	3 (6)	2 (6)	1 (6)^b^
Post‐operative complications^c^			
Same day visit only (ED/medical day unit)	5 (9)	4 (11)	1 (6)
Readmission	2 (4)	1 (3)	1 (3)
Acute chest syndrome	0	0	0
Infection	0	0	1 (6)

Abbreviations: EBL, estimated blood loss; SNM, surgical management of miscarriage.

^a^ Percentages are calculated as percentages of total procedures.

^b^ This was a planned transfusion according the patient's regular automated exchange transfusion schedule.

^c^ Defined as any encounter with a healthcare professional within 28 days of the procedure.

Following the procedure, 40 patients (75%) were discharged the same day or the following day. Eight (15%) were admitted for one night, five for observation only, two for post‐operative pain and one for a post‐operative transfusion following a repeat Hb of 66 g/L. Five patients (9%) were admitted for periods longer than one night (range 2–8, median 3 nights), four for post‐operative pain, with concomitant urinary tract infection and one for psychiatric concerns. Of 13 post‐procedural admissions, nine had the genotype HbSS, while four had genotype HbSC. There was no correlation between prior transfusion and being admitted for at least one night post‐operatively (Fisher's exact test, *p* = 0.5). Three patients were transfused post‐operatively: one described above, one who remained an inpatient and was transfused for post‐operative pain and one who remained an inpatient but had a planned automated red cell exchange transfusion according to her usual schedule.

Post‐operative complications were defined as an encounter with a healthcare provider within 28 days. There were no cases of post‐operative acute chest syndrome and no reported post‐operative surgical complication. Seven patients (13%) re‐presented to the hospitals within 28 days of the procedure; of these, 5 (9%) had a same‐day visit to the emergency department (ED) or medical day unit (MDU), all for pain, and 2 (4%) were re‐admitted to hospital overnight, one for severe pain and one for urinary tract infection. Of all six patients re‐presenting with pain, this was classified as ‘sickle cell‐related pain’ in five cases and anxiety‐related in one. Exploratory analysis revealed that post‐operative complications occurred in 2/14 (14%) of patients who received a pre‐operative transfusion and 5/39 (13%) of patients who did not receive a pre‐operative transfusion. There was no significant difference between groups (Fisher's exact test, *p* = 1.00).

The results of this multicentre study reveal differing outcomes in a UK cohort of SCD patients undergoing surgical evacuation of the uterus, compared to recent US data. While transfusion rates were similar (31% US vs. 26% UK), we note a higher rate of post‐operative re‐presentation to the ED (25% vs. 13%) and re‐admission (16% vs. 4%) in the larger US study.^9^ This may be because their cohort did not have any direct post‐operative admissions, and differing healthcare models may account for this, such as difficulty directly admitting a patient from a clinic setting. We also note the much higher rate of pre‐existing acute chest syndrome (45% vs. 4%) which may contribute to this although the total number of reports of acute chest syndrome were low in both studies (2/71 vs. 0/53).

Since the publication of the TAPS study, transfusion prior to low‐ and medium‐risk procedures has become commonplace for patients with SCD. As there were no cases of surgical abortion or SMM in the original paper, local practice has largely been determined by individual clinicians and there is no unifying guidance despite how common these procedures are in young women. In particular, there is no mention of how to manage abortion or miscarriage in either the British Society of Haematology nor the WHO guidelines of management of pregnancy in women with SCD.^11^, ^12^ While transfusion is widely available and generally safe, it is not without risks. Long term complications of transfusion, such as allo‐antibody formation, may result in significant consequences such as transfusion reactions or hyperhaemolysis syndrome.

This service evaluation suggests that, while surgical evacuation of the uterus can be carried out safely without transfusion in most cases, this is based on small sample sizes, in both this and previous work. We acknowledge potential selection bias, as patients felt most likely to have complications may be more likely to receive transfusions, and we must therefore exercise caution when reviewing data around pre‐operative transfusion. It is possible that some post‐operative complications have not been included in our data centre if patients presented to another centre, although as haemoglobinopathy care in the United Kingdom is centred around regional networks, we believe the risks of this to be low. [Correction added on 7 April 2026, after first online publication: The preceding sentence has been updated to include the word “not” for clarity.] A larger, randomised controlled trial would help confirm that transfusion‐free surgical abortion/SMM is safe in all but the highest risk patients.

We suggest that, when future local or national policies for managing SCD patients for surgical abortion/SMM, procedures should be conducted in a generalised hospital setting, ideally with a specialised haemoglobinopathy team unless the urgency of the procedure precludes this. Second, procedures should be discussed by the gynaecology team with the haematology team and a multidisciplinary decision made around transfusion, taking into consideration prior medical and transfusion history. Third, we would suggest that all patients have a full blood count prior to the procedure and we would consider proceeding without prior transfusion unless: (a) there is a prior history of acute chest syndrome, (b) there is a significant drop in haemoglobin to less than 60 g/dL or more than 20 g/dL below baseline and (c) there are gynaecological reasons where bleeding may be excessive or the duration of anaesthesia lasts beyond 1 h. For patients with difficult transfusion requirements such as multiple allo‐antibodies, units may be ordered pre‐emptively but do not necessarily need to be transfused. If transfusion is deemed necessary, the minimum number of units required should be given, with one unit transfused initially.

In summary, this is the first European paper of its kind to examine surgical abortion and SMM in SCD and is suggestive that transfusion prior to such procedures is not required in most cases. We hope that this guides future practice and can be used to determine further local and national guidelines.

## AUTHOR CONTRIBUTIONS

S Wolf, T Masters and A Andreeva conceived the study. S Wolf, N Lewis, F Oyesanya, K Birthwright and A Andreeva collected the data. S Wolf wrote the paper, and all authors reviewed the final manuscript.

## FUNDING INFORMATION

No funding was required for this study.

## CONFLICT OF INTEREST STATEMENT

The authors have no conflicts of interest to disclose.

## ETHICAL STATEMENT

This study was carried out as part of a service evaluation of existing services at both sites and was approved by Barts Health and Homerton institutional quality improvement processes. In line with Health Research Authority guidance, it did not constitute research and, therefore, did not require individual consent. Data were anonymised and handled in accordance with local governance standards.

## Data Availability

The data that support the findings of this study are available on request from the corresponding author. The data are not publicly available due to privacy or ethical restrictions.
